# New Method of Papillectomy May Decrease Recurrence: Anchoring Method versus Conventional Method

**DOI:** 10.3390/jcm13113226

**Published:** 2024-05-30

**Authors:** Jonghyun Lee, Yong Bo Park, Sung Yong Han, Dong Chan Joo, Seung Min Hong, Kiyoun Yi, Dong Uk Kim

**Affiliations:** 1 Division of Gastroenterology, Biomedical Research Institute, Pusan National University Hospital, Busan 49241, Republic of Korea; keiasikr@nate.com (J.L.); gyongbo@naver.com (Y.B.P.); mirsaint@hanmail.net (S.Y.H.); asclllepios@gmail.com (D.C.J.); lucky77i@naver.com (S.M.H.); yikiyoun@hanmail.net (K.Y.); 2 Department of Internal Medicine, College of Medicine, Pusan National University, Yangsan 50612, Republic of Korea; 3 Department of Internal Medicine, CHA Gumi Medical Center, CHA University, Gumi-si 39295, Republic of Korea

**Keywords:** ampulla of Vater, adenoma, cholangiopancreatography, endoscopic retrograde

## Abstract

**Background/Objectives**: Endoscopic papillectomy (EP) is the preferred treatment for ampullary tumors because it has fewer side effects than surgical removal. This study retrospectively compared a new anchoring EP method (A-EP) with the conventional (C-EP) approach. **Methods**: Ninety-nine patients who underwent EP at a single medical institution between 2009 and 2021 were retrospectively reviewed. In all patients, the indications for EP were pathological adenoma with <10 mm of biliary invasion and a tumor diameter <30 mm on endoscopic ultrasonography. The exclusion criteria were antiplatelet/anticoagulant use, previous upper GI surgery, or prior biliary/pancreatic endoscopic therapy. One expert endoscopist performed the two types of EPs, A-EP and C-EP. **Results**: Sixty-two patients underwent A-EP, and 37 underwent C-EP. There were no significant differences in baseline characteristics, such as sex, age, tumor size, and ductal invasion on endoscopic ultrasound. The A-EP group had higher en bloc resection rates (95.2% vs. 78.4%, *p* = 0.010). Although the difference was not statistically significant, it tended towards fewer incidences of pancreatitis (*p* = 0.081) and duct stricture (*p* = 0.081). The recurrence rate was lower in the A-EP group (8.1% vs. 37.8%, *p* = 0.000). There were no significant differences between the two groups regarding the follow-up period (A-EP vs. C-EP, 725 vs. 1045 days, *p* = 0.109) or the days of recurrence (A-EP vs. C-EP, 341 vs. 562 days, *p* = 0.551). **Conclusions**: A-EP showed better outcomes than C-EP in terms of en bloc resection and recurrence rates, providing evidence for the efficacy of this novel EP method.

## 1. Introduction

Ampullary tumors are rare and account for only 0.2% of gastrointestinal tumors [1,2]. These tumors arise from the ampulla of Vater, an important anatomical landmark where the bile duct and the pancreatic duct meet and empty into the duodenum. An ampullary adenoma is a dysplasia that occurs in and around the duodenal papilla [3,4]. It represents a significant precancerous condition that necessitates clinical attention. The prevalence of ampulla adenoma ranges from 0.04 to 0.12%. When these adenomas progress into malignant ampullary tumors, the prognosis becomes serious, with a 5-year survival rate between 38.8% and 47.2% [1,5].

Given their potential to progress to adenocarcinoma, ampullary adenomas are considered precancerous lesions that require complete resection to prevent malignant transformation [6,7,8]. There are two treatment methods for ampullary adenomas: endoscopic papillectomy (EP) and surgical ampullectomy. According to a meta-analysis, there is no significant difference in long-term recurrence rates between these two methods [9]. However, for lesions smaller than 20 mm to 30 mm where en-bloc resection is feasible, endoscopic procedures are generally preferred due to their minimally invasive nature and lower incidence of complications compared to surgical removal [10,11,12,13,14]. It is important to note that both endoscopic resection and surgical treatment should be performed by experienced and well-trained medical staff and centers to achieve high rates of complete resection and low recurrence rates.

Nevertheless, according to previously reported research, the complete resection rate was 77.7%, and the recurrence rate was 12.6% for EP for ampullary adenoma [10,15,16,17,18,19,20]. To increase the complete resection rate and reduce the complication and recurrence rates, several studies have evaluated the efficacy of pancreatic duct stent placement and argon plasma coagulation after resection. These methods aim to increase the complete resection rate and minimize complications and recurrence. However, the best method has not been identified and recommended by experts [3,21,22,23,24,25,26,27,28,29,30].

One of the challenges in conventional endoscopic procedures (C-EP) is the slippage of the snare from the intended lesion, which complicates the procedure and can reduce its effectiveness. A previous study demonstrated that using a balloon as an anchor to stabilize the endoscopic scope could enhance the resection rate [31]. Based on these results, our institution developed and implemented a technique using needle-knife fistulotomy to create an anchoring site, thereby facilitating endoscopic resection.

**Figure 1:**
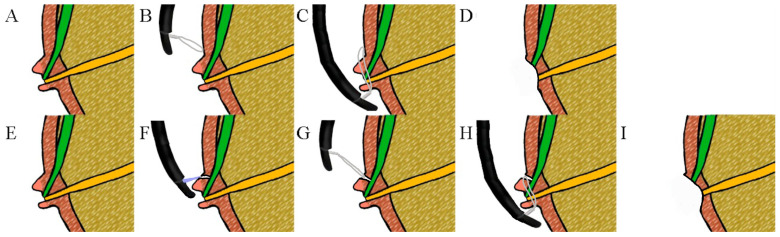
Two methods of endoscopic papillectomy (EP). (**A**–**D**) Conventional EP (C-EP); (**A**) Ampullary adenoma; (**B**) Snare tip placed at the upper edge of the ampulla; (**C**) Snare is placed around the lesion; (**D**) Papillectomy done using C-EP; (**E**–**I**) Anchoring EP (A-EP); (**E**) Ampullary adenoma; F: Needle-knife fistulotomy; (**G**) Snare tip anchored to the fistulotomy site; (**H**) Snare placed around the lesion; (**I**) Papillectomy performed by A-EP.

In this study, the usefulness of anchoring EP (A-EP) was retrospectively compared with that of conventional EP (C-EP). A-EP is a modification of endoscopic mucosal resection that incorporates precutting, a technique designed to address the challenge of removing large lesions that are difficult to resect endoscopically by anchoring the snare tip for lesions in the general mucosa [32]. A-EP creates a fistula on the roof of the oral side of the tumor and prevents the snare from slipping by fixing the end of the noose to the fistula site when grasping the lesion (Figure 1). Furthermore, A-EP facilitates biliary cannulation after resection. Therefore, A-EP has the potential to excise lesions more effectively than C-EP.

The primary objective of this study was to determine whether A-EP could achieve higher complete resection rates and lower complication and recurrence rates compared to C-EP. By providing a detailed comparison of these two techniques, we aim to identify the most effective approach for the treatment of ampullary adenomas, ultimately improving patient outcomes in these challenging clinical lesions.

## 2. Materials and Methods

### 2.1. Study Design

This study enrolled 99 patients who underwent A-EP or C-EP after the detection of an ampullary adenoma through upper endoscopy at Pusan National University Hospital from May 2009 to July 2021. The study was conducted retrospectively to confirm the recurrence rate for each mucosal resection method based on the test results. In all patients, endoscopic mucosal resection was performed when the pancreatic duct or biliary tract infiltration was less than 1 cm, the tumor diameter was less than 30 mm, and the adenoma was histologically confirmed by biopsy [31,33]. The exclusion criteria were a history of upper gastrointestinal surgery, previous biliary/intrapancreatic therapy, and antiplatelet or anticoagulant use that could not be discontinued. The procedures, biopsy results, and progress, except for the patient’s personal information, were confirmed through the medical records. This study was conducted in accordance with the ethical guidelines of the Declaration of Helsinki (2013 revision) and approved by the Research Ethics Review Committee of Pusan National University Hospital. (IRB No. 2305-016-127, 23 May 2023) This study was conducted retrospectively based on already accumulated data. Since the data provided did not include any personally identifiable information, the risk to the study subjects was minimal, and, thus, informed consent was waived and approved by the committee.

**Figure 2:**
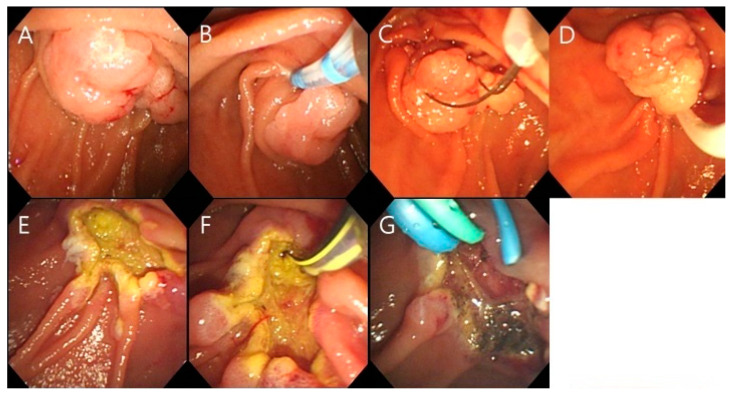

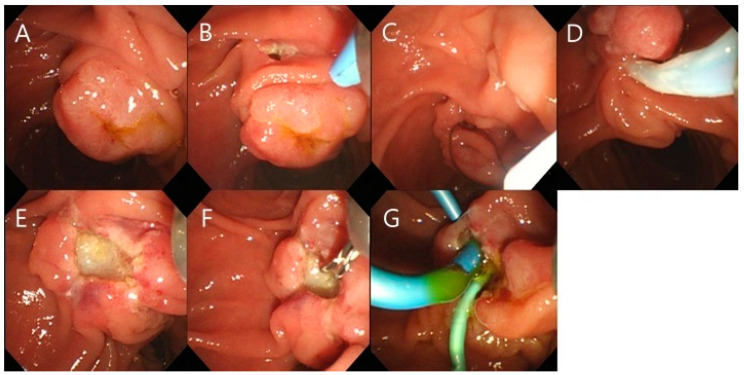
Anchoring method of endoscopic papillectomy done on two patients. (**A**) Ampullary adenoma; (**B**) Needle knife fistulotomy; (**C**) The snare tip was anchored to the fistulotomy site; (**D**) Snare papillectomy was performed (the snare tip is on the upper edge during fistulotomy); (**E**) Upper margin of papillectomy coincides with the upper edge of fistulotomy before papillectomy; (**F**) Cannulation of the bile and pancreatic ducts; (**G**) Endoscopic insertion of retrograde biliary and pancreatic drainage plastic stents.

A-EP is a technique used to create a fistula on the roof of the oral side of the tumor to prevent slippage when using a snare and facilitate biliary insertion after resection. The detailed A-EP procedure carried out is as follows: First, bile duct cannulation was performed using needle knife fistulotomy. Second, the end of the snare was exposed approximately 1–2 mm from the catheter and fixed to the fistula site. Third, the snare was pushed gently toward the fistula while keeping it wide open. Finally, the lesion was widely entrapped, and the tumor was resected completely with a snare (Figure 2). All EPs were performed by an expert endoscopist. From 2009 to 2015, C-EP was the preferred treatment method for patients. A mixed approach utilizing both C-EP and A-EP was employed between 2016 and 2017. After 2018, the preference shifted towards the exclusive use of A-EP.

### 2.2. Study Outcome Measures

The sex and age of the patients and their endoscopic ultrasound (EUS) findings (ductal invasion) and lesion locations (major ampulla) were also investigated. The lesion size, marginal area infiltration, biliary tract infiltration, and complete resection were reviewed by the pathological reports after the procedure. We evaluated whether endoscopic retrograde pancreatic drainage (ERPD) insertion and prophylactic argon plasma coagulation (APC) were performed to prevent adverse events such as pancreatitis or bleeding. Complications after the procedure, such as bleeding (a drop of Hb 2 or more, confirmed by follow-up endoscopy due to suspicious bleeding such as melena), hyperamylasemia (>128 U/L compared with blood test results before and after the procedure), pancreatitis (typical abdominal pain, three-fold increase in pancreatic enzyme levels or characteristic imaging results), biliary and pancreatic duct stenosis (hepatic or pancreatic blood test abnormalities with stenosis found on endoscopic retrograde cholangiopancreatography [ERCP], computed tomography [CT], or magnetic resonance cholangio pancreatography), perforation (confirmed by imaging tests), and the number of days in the hospital were checked and compared. The date of observation, recurrence rate (histological confirmation), treatment method for recurrence (APC, EP, surgery, refusal of surgery), and presence or absence of secondary recurrence (histological confirmation) for each treatment method were compared.

### 2.3. Statistical Analysis

Statistical analyses were carried out using SPSS statistical software (version 22.0, IBM Corp., Armonk, NY, USA). The analysis encompassed both categorical and continuous data to provide a comprehensive understanding of the study results. For categorical data, differences between groups were summarized in terms of frequency and percentage, utilizing the chi-square test to assess statistical significance. This approach allowed for the evaluation of proportions and distributions within the data set, facilitating a clear comparison between the two groups under study.

For continuous variables, the differences between the two groups were analyzed using an independent *t*-test. This method enabled the determination of whether there were statistically significant differences in the means of these variables between the groups. The results were summarized as mean ± standard deviation, providing a detailed overview of the central tendency and variability within the data. This rigorous approach ensured that the comparisons made were robust and reliable.

Furthermore, multivariate analysis was conducted to account for the potential influence of multiple variables simultaneously. This analysis included variables that were identified as having low *p*-values in the univariate analysis. By doing so, the study aimed to identify independent predictors and control for confounding factors, offering a deeper insight into the relationships between the variables. The threshold for statistical significance was set at *p* < 0.05, ensuring that the findings were robust and statistically meaningful. This comprehensive statistical approach allowed for a thorough examination of the data, supporting the study’s conclusions with solid empirical evidence.

## 3. Results

Information on the 99 patients is summarized in Table 1. Sixty-two patients underwent A-EP, and 37 underwent C-EP. The proportions of males in the A-EP and C-EP groups were 62.9% and 59.5%, respectively. At the time of ampullary adenoma diagnosis, the mean age of each group was 64.8 years and 64.4 years, respectively, and no statistically significant difference was observed. There was no significant difference in biliary duct or pancreatic duct invasion of <10 mm (7.7% vs. 6.5%, *p* = 0.814) on EUS performed for preoperative lesion evaluation.

Postoperative ERPD insertion (43 (69.4%) vs. 31 (80.5%), *p* = 0.112) and prophylactic APC (32 (51.6%) vs. 19 (51.4%], *p* = 0.980)) was not significantly different between the two groups. There were no differences between the two groups in adverse events such as bleeding (45 (72.6%) vs. 25 (65.9%), *p* = 0.600), hyperamylasemia (28 (45.2%) vs. 15 (40.5%)), pancreatitis (9 (14.5%) vs. 6 (16.2%), *p* = 0.934), perforation (0 (0%) vs. 2 (2.7%), *p* = 0.081), pancreatic bile duct stenosis (0 (0%) vs. 2 (5.4%), *p* = 0.081), and the number of days hospitalized (11.3 ± 4.4 vs. 11.9±5.6). After the procedure, the histologically confirmed lesion size (1.02 ± 0.51 vs. 1.21 ± 0.73, *p* = 0.137), the bile duct infiltration (3.2% vs. 9.8%, *p* = 0.075), and the margin invasion were also not significantly different between the two groups. There was a statistically significant difference in complete resection rates (59 (95.2%) vs. 29 (78.4%), *p* = 0.010), as confirmed by histological examination after the procedure in each group.

The multivariate analysis of procedural outcomes based on A-EP and C-EP is summarized in Table 2. This analysis included several variables, including mass size, bile duct invasion, ERPD insertion, en bloc resection, and adverse events, all of which had relatively low P values. The average mass size was slightly smaller in the A-EP group (1.02 ± 0.51 cm) compared to the C-EP group (1.21 ± 0.73 cm), though this difference was not statistically significant (*p* = 0.347). Bile duct invasion was observed in 3.2% and 10.8% of cases in the A-EP and C-EP groups, respectively (*p* = 0.225). A higher percentage of ERPD insertions was noted in the C-EP group (83.8%) than in the A-EP group (69.4%; *p* = 0.198). It was also not statistically significant. A significant difference was observed in the en bloc resection rates, with 95.2% in the A-EP group and 78.4% in the C-EP group (*p* = 0.032). The relative risk of en bloc resection in the A-EP group was substantially higher at 4.943 (95% confidence interval [CI]: 1.143−21.369), indicating a statistically significant advantage of A-EP in achieving en bloc resection. The incidence of adverse events was slightly higher in the A-EP group (93.5%) than in the C-EP group (86.5%; *p* = 0.603). No significant differences were found between the two groups. En-bloc resection was the most significant factor in both the univariate and the multivariate analyses.

These findings indicate that although both A-EP and C-EP exhibit comparable safety profiles, A-EP appears to have a notable advantage in terms of en bloc resection rates. This is particularly significant because en bloc resection is critical for ensuring complete removal of lesions, which can affect long-term patient outcomes. The statistically significant difference observed suggests that A-EP could improve clinical outcomes by minimizing the likelihood of residual disease.

The prognosis for each treatment method is summarized in Table 3. The number of days of follow-up (725.4 ± 919.0 vs. 1095.4 ± 1070.5, *p* = 0.064) had no significant difference, but the recurrence rate (5 (8.1%) vs. 14 (39.0%), *p* = 0.000) was significantly lower in A-EP than in C-EP. The days until the first relapse (341.0 ± 198.5 vs. 609.7 ± 569.1, *p* = 0.442) also had no significant difference. Treatment method of the first recurrent lesion included ablation with APC (0 (0%) vs. 8 (50%)), re-endoscopic mucosal resection using a snare (3 (60%) vs. 3 (21.4%)), surgery (1 (10%) vs. 2 (14.3%)) and refusing surgery (1 (10%) vs. 1 (7.3%)) for A-EP and C-EP, respectively. In each group, there was no significant difference in the second recurrence rate after treatment for the first recurrence (2/5 (40%) vs. 4/14 (28.6%); *p* = 0.659).

## 4. Discussion

In this study, the A-EP group had higher complete resection rates and lower recurrence rates than the C-EP group. Complete endoscopic resection of ampullary adenomas is emphasized because ampullary adenomas are known to be precursors to ampullary cancer [6,7,8]. Typically, complete resection is defined as the absence of endoscopic-looking and histologically proven residual adenomas during a follow-up period of 3−6 months after undergoing resection through EP [34].

Several studies have investigated methods for increasing the complete resection rate and reducing the recurrence rate of papillary adenomas. According to the results of these studies, recurrence is related to several factors, including tumor size, intraductal invasion, and en bloc resection (Table 4). Among them, en bloc resection is the most frequently suggested treatment. With the recent development of endoscopes and accessory tools, the rates of utilizing en bloc resection have increased, and the role of endoscopes in ampullary adenomas is gradually expanding.

During C-EP, the snare has a high possibility of sliding, contrary to the operator’s intention, owing to the ampullary roof. In addition, the last field of view looks down at the bottom of the lesion when the snare captures the elevated lesion. Since the view of the lesion to be resected is limited, there is a high probability that the lesion will be cut without being fully captured. However, during A-EP, the snare was fixed as intended by the surgeon after the fistulotomy. Thus, the problems of blindness in the upper part of the lesion and slipping of the snare were resolved. Thus, the uppermost part of the lesion could be trapped using a snare.

In this study, the en bloc resection rate (59 (95.2%) vs. 29 (78.4%), *p* = 0.010) was significantly higher in the A-EP group, and the recurrence rate (5 (8.1%) vs. 14 (37.8%), *p* = 0.000) was also lower. This can be considered the result of refining the disadvantages of C-EP while maintaining the advantages confirmed in other studies [38,39]. In addition, considering that 6.9−43.8% of ampullary adenomas in other studies were accompanied by infiltration into the bile duct or pancreatic duct, the recurrence rate (8.1%) of the A-EP group in this study could indicate complete resection in almost all patients [12,15,21,22,40]. The incidence of perforation and pancreatic and bile duct stenosis post-procedure showed a *p*-value of 0.081. Although not statistically significant, no adverse events were observed using the A-EP method. As mentioned previously, this can be attributed to the use of anchoring, which prevents slippage and allows precise placement of the snare. This technique reduces perforation rates and improves en bloc resection rates. This approach contributes to lower recurrence rates and better outcomes.

Therefore, the results of the present study are significant. The margin positivity rate (16 (39.0%) vs. 11 (40.3%), *p* = 0.896) was similar in both groups; however, the recurrence rate was lower in the A-EP group (5 (8.1%) vs. 14 (39.0%), *p* = 0.000). This may be because the evaluation of the margins was not accurate owing to the cautery effect.

The mean size of resected lesions was smaller for A-EP than for C-EP (1.02 cm ± 0.51 cm vs. 1.21 cm ± 0.73 cm). This may be because the A-EP samples were larger than the C-EP samples (62 vs. 37), and the lesion size of patients who underwent C-EP before attempting A-EP was relatively large (up to 3 cm). However, as this difference was not statistically significant, it was thought to have little effect on the results of this study.

This study had several limitations. First, the sample size was small, and the study was conducted at a single institution. Therefore, a multicenter follow-up study with a larger number of patients is required. Second, because this study was retrospective, caution is required when interpreting the data. For A-EP, the procedure was improved based on the results of another study that used a balloon as the anchor. Based on these results, a prospective study is planned for future implementation. Third, as there have been relatively recent attempts at A-EP, the follow-up period after the procedure was shorter in the A-EP group than in the C-EP group. This may have caused a difference in the recurrence rates. However, in both groups, the average recurrence time was approximately 24 months, and follow-up was performed for an average of 720 days. Considering that other previously reported results showed that recurrence occurred within 14 months after the procedure, the number of follow-up days used to compare the recurrence rates in the A-EP and C-EP groups in this study is considered sufficient [21,41] and is expected to have little impact on the results of this study.

## 5. Conclusions

In conclusion, this study demonstrates that A-EP offers significant advantages over C-EP for the treatment of ampullary adenomas. A-EP showed higher rates of complete en bloc resection and lower recurrence rates, which are crucial for preventing the recurrence of these adenomas into malignant ampullary tumors. The technique of using needle-knife fistulotomy to anchor the snare significantly improved the stability and precision of the procedure, reducing the likelihood of snare slippage and ensuring more complete removal of the lesions.

Despite the study’s limitations, including its retrospective nature and the relatively small sample size from a single institution, the findings suggest that A-EP is a more effective and safer method for the resection of ampullary adenomas. The higher en bloc resection rates achieved with A-EP directly correlate with lower recurrence rates, underscoring the importance of complete resection in these cases. The absence of significant differences in adverse events between A-EP and C-EP further supports the safety profile of A-EP.

Future studies, particularly multicenter prospective trials with larger patient cohorts and longer follow-up periods, are warranted to confirm these findings and further refine the A-EP technique. Additionally, ongoing evaluation and optimization of adjunctive therapies, such as pancreatic duct stenting and prophylactic argon plasma coagulation, should be pursued to enhance the efficacy of endoscopic treatments for ampullary adenomas.

Overall, this study provides valuable insights into improving clinical outcomes for patients with ampullary adenomas and highlights the potential of A-EP as a preferred endoscopic treatment method.

## Figures and Tables

**Table 1:** Baseline characteristics and results of both procedures.

	A-EP (n = 62)	C-EP (n = 37)	*p*-Value
Sex, male (%)	39 (62.9)	22 (59.4)	0.736
Age (years)	64.8 ± 11.6	65.3 ± 12.7	0.870
EUS finding B-inv or p-inv	3/39 (7.7)	1/15 (6.7)	0.814
Mass size (cm)	1.02 ± 0.51	1.21 ± 0.73	0.137
B-duct invasion	2 (3.2)	4 (10.8)	0.075
ERPD insertion	43 (69.4)	31 (83.8)	0.112
Prophylactic APC	32 (51.6)	19 (51.4)	0.980
Margin+	16 (39.0)	11 (29.7)	0.896
Margin free/lat+/ver+/both/un-clear	37(59.7)/9(14.5)/2(3.2) /5(8.1)/9(14.5)	23(62.2)/6(16.2)/2(5.4) /3(8.1)/3(8.1)	0.519
En-bloc resection	59 (95.2)	29 (78.4)	0.010 *
Adverse events	58 (93.5)	32 (86.5)	0.157
Bleeding	45 (72.6)	25 (67.6)	0.600
Hyperamylasemia	28 (45.2)	15 (40.5)	0.507
Post-ERCP pancreatitis	9 (14.5)	6 (16.2)	0.934
Perforation	0 (0)	2 (5.4)	0.081
Duct stricture	0 (0)	2 (5.4)	0.081
Admission days	11.3 ± 4.4	11.9 ± 5.6	0.594

Abbreviations: A-EP, anchoring endoscopic papillectomy; C-EP conventional endoscopic papillectomy; ERPD, endoscopic retrograde pancreatic drainage; ERCP, endoscopic retrograde cholangiopancreatography; APC, argon plasma coagulation; *, Statistically significant

**Table 2:** Multivariate analysis of results of both procedures.

	A-EP (n = 62)	C-EP (n = 37)	*p*-Value	Relative Risk (95% CI)
Mass size (cm)	1.02 ± 0.51	1.21 ± 0.73	0.347	0.707 (0.343−1.456)
B-duct invasion	2 (3.2)	4 (10.8)	0.225	0.389 (0.085−1.787)
ERPD insertion	43 (69.4)	31 (83.8)	0.198	0.484 (0.160−1.463)
En-bloc resection	59 (95.2)	29 (78.4)	0.032 *	4.943 (1.143−21.369)
Adverse events	58 (93.5)	32 (86.5)	0.603	0.678 (0.156−2.941)

Abbreviations: A-EP, anchoring endoscopic papillectomy; C-EP, conventional endoscopic papillectomy; CI, confidence interval; ERPD, endoscopic retrograde pancreatic drainage; *, Statistically significant

**Table 3:** Prognosis of each papillectomy method.

	A-EP (n = 62)	C-EP (n = 37)	*p*-Value
Follow-up days	725.4 ± 919.0	1045.8 ± 1010.3	0.109
Recurrence rate	5 (8.1)	14 (37.8)	0.000 *
Recurrence days	341.0 ± 198.5	562.1 ± 603.1	0.551
Method of treating recurrence (APC/snare/op/op refuse)	0(0)/3(60)/1(10)/1(10)	8(57.1)/3(21.4)/2(14.3)/1(7.3)	0.158
Recurrence after first recurrence management	2/5 (40)	4/14 (28.6)	0.659

Abbreviations: APC, argon plasma coagulation; *, Statistically significant

**Table 4:** Factors associated with recurrence after papillectomy.

Ref.	Year	Number of Patients	Related Factor	*p*-Value
Ridtitid, Wiriyaporn, et al. [11]	2014	182	No en-bloc resection	<0.0001
Bohnacker, Sabine, et al. [35]	2005	106	Intraductal invasion	<0.001
Ahn, Dong-Won, et al. [36]	2013	43	No en-bloc resection	0.007
Gondran, Hannah, et al. [37]	2022	277	Tumor size > 2 cm	0.003
			Lateral extension	0.021
			Intra ductal invasion	0.001
			No en-bloc resection	0.006
Li, Shuling, et al. [17]	2018	110	No en-bloc resection	0.001

## Data Availability

All data used in this study are provided in this article.
